# *Porphyromonas gingivalis* oral infection exacerbates the development and severity of collagen-induced arthritis

**DOI:** 10.1186/ar4376

**Published:** 2013-11-12

**Authors:** Julie Teresa Marchesan, Elizabeth Ann Gerow, Riley Schaff, Andrei Dan Taut, Seung-Yun Shin, James Sugai, David Brand, Aaron Burberry, Julie Jorns, Steven Karl Lundy, Gabriel Nuñez, David A Fox, William V Giannobile

**Affiliations:** 1 Department of Periodontics and Oral Medicine, University of Michigan School of Dentistry, Ann Arbor, MI, USA; 2 Department of Periodontology, Institute of Oral Biology, School of Dentistry, Kyung Hee University, Seoul, South Korea; 3 Research Service, Veterans Affairs Medical Center, Memphis, TN, USA; 4 Department of Pathology, University of Michigan Medical School, Ann Arbor, MI, USA; 5 Department of Internal Medicine – Division of Rheumatology, University of Michigan Medical School, Ann Arbor, MI, USA

## Abstract

**Introduction:**

Clinical studies suggest a direct influence of periodontal disease (PD) on serum inflammatory markers and disease assessment of patients with established rheumatoid arthritis (RA). However, the influence of PD on arthritis development remains unclear. This investigation was undertaken to determine the contribution of chronic PD to immune activation and development of joint inflammation using the collagen-induced arthritis (CIA) model.

**Methods:**

DBA1/J mice orally infected with *Porphyromonas gingivalis* were administered with collagen II (CII) emulsified in complete Freund’s adjuvant (CFA) or incomplete Freund’s adjuvant (IFA) to induce arthritis. Arthritis development was assessed by visual scoring of paw swelling, caliper measurement of the paws, mRNA expression, paw micro-computed tomography (micro-CT) analysis, histology, and tartrate resistant acid phosphatase for osteoclast detection (TRAP)-positive immunohistochemistry. Serum and reactivated splenocytes were evaluated for cytokine expression.

**Results:**

Mice induced for PD and/or arthritis developed periodontal disease, shown by decreased alveolar bone and alteration of mRNA expression in gingival tissues and submandibular lymph nodes compared to vehicle. *P. gingivalis* oral infection increased paw swelling and osteoclast numbers in mice immunized with CFA/CII. Arthritis incidence and severity were increased by *P. gingivalis* in mice that received IFA/CII immunizations. Increased synovitis, bone erosions, and osteoclast numbers in the paws were observed following IFA/CII immunizations in mice infected with *P gingivalis*. Furthermore, cytokine analysis showed a trend toward increased serum Th17/Th1 ratios when *P. gingivalis* infection was present in mice receiving either CFA/CII or IFA/CII immunizations. Significant cytokine increases induced by *P. gingivalis* oral infection were mostly associated to Th17-related cytokines of reactivated splenic cells, including IL-1β, IL-6, and IL-22 in the CFA/CII group and IL-1β, tumor necrosis factor-α, transforming growth factor-β, IL-6 and IL-23 in the IFA/CII group.

**Conclusions:**

Chronic *P. gingivalis* oral infection prior to arthritis induction increases the immune system activation favoring Th17 cell responses, and ultimately accelerating arthritis development. These results suggest that chronic oral infection may influence RA development mainly through activation of Th17-related pathways.

## Introduction

Periodontal disease (PD) is an immune-inflammatory infection of the tooth-supporting structures. The disease affects one-half of the US population over 30 years of age and is the major cause of tooth loss among adults [1]. For PD to develop, a microbial shift must occur from a normally symbiotic microbiota into a dysbiotic state [2]. While this exact shift is still being determined, some key bacteria are consistently shown to be important for PD. *Porphyromonas gingivalis* is a Gram-negative pathogenic bacterium associated with increased risk of periodontal breakdown and disease recurrence [3]. In addition, *P. gingivalis* has been recently indicated as a keystone pathogen of disease-provoking periodontal microbiota [2]. *P. gingivalis* activates several innate immune receptors, including toll-like receptor-2, toll-like receptor-4, nucleotide-binding oligomerization domain-2, and protease-activated receptor-2, which ultimately contribute to disease initiation and progression [4-6]. Classically, periodontitis is considered a mixed T-helper type (Th)1/Th2-driven disease, with a Th1 cytokine profile being the major mediator in the early/stable lesion and a dominance of a Th2 cells in the advanced/progressive lesion [7]. The role of Th17 cells in periodontitis is still under investigation, with various lines of evidence suggesting that it can either drive or protect against disease development [8,9]. While the effect of *P. gingivalis* and the role of cytokines in inflammation of the oral tissues have been explored, only a few preclinical studies have evaluated the systemic effect of periodontitis [10,11] and how it may affect the development of other diseases in preclinical models. The bidirectional association of periodontitis with other diseases, including cardiovascular disease [12], diabetes mellitus [13], and rheumatoid arthritis (RA) [14], underscores the relevance of understanding the cytokine networks implicated in such associations.

RA is a chronic inflammatory autoimmune disease that affects 1% of the population [15]. A complex cytokine network is directly involved in specific immunological processes that promote autoimmunity, chronic inflammation, and ultimately tissue destruction in RA [16]. Cytokine modulation therapies, such as anti-tumor necrosis factor (TNF) alpha, interleukin (IL)-6R, anti-IL-23p19, and anti-IL-22 are shown to alter disease development in preclinical and/or clinical settings [16-20]. Understanding the complex cytokine milieu that develops in all stages of RA is therefore crucial for identifying potential treatments for patients [16].

Accumulating clinical evidence supports a bidirectional association between periodontitis and RA in the clinical setting [14,21,22]. Some clinical studies suggest a direct effect of periodontal disease in established RA by decreased serum erythrocyte sedimentation rate, C-reactive protein, TNFα levels and improved Disease Activity Score in 28 joints after periodontal treatment is provided to RA patients [23-25]. Although the effect of periodontal treatment in RA needs to be confirmed in larger, controlled trials, these results suggest a direct effect of periodontal disease in RA. In addition, successful treatment of RA patients with antibiotics against bacterial anaerobic infections suggests the involvement of bacteria in the etiopathogenesis of RA [26]. Only one report has shown that prior *P. gingivalis* oral infection augments development of collagen antibody-induced arthritis in mice [27]. While analysis of C-reactive protein indicates that inflammation is a main player in the additional effect observed, no further cytokine analysis was performed. One very useful model for studying RA is collagen-induced arthritis (CIA) in rodents, which has not been explored in association with periodontitis. Since both CIA and PD are inflammatory and Th-driven diseases, an improved understanding of the effect of chronic PD on the immune activation of arthritis would be of value.

The present study was performed to determine the role of *P. gingivalis* oral infection in modulating Th cell-driven responses and arthritis development in CIA. Our results indicate that *P. gingivalis* oral infection augmented the innate immune response during arthritis development. Our data demonstrate that mice infected with *P. gingivalis* displayed increased Th17-driven responses in the serum via IL-17 and IFNγ, reactivated splenocytes via IL-1β, IL-6, TNFα, transforming growth factor beta (TGF-β), and IL-23, increased osteoclast numbers in the joints, and enhanced arthritis progression and development.

## Methods

### Study design

DBA1/J male mice (Jackson Laboratory, Bar Harbor, ME, USA), 6 weeks old, were infected with *P. gingivalis* for 15 days (D0 to D15) and immunized 15 days later (D30) with collagen II (CII) emulsified in either complete Freund’s adjuvant (CFA) or incomplete Freund’s adjuvant (IFA). Mice were sacrificed at baseline (D0), D30 (before arthritis induction), D44 (14 days after arthritis induction), and D73 (43 days after arthritis induction). All animal experiments were approved by the Institutional Animal Care and Use Committee of the University of Michigan (Ann Arbor, MI, USA) and conformed to ARRIVE guidelines for preclinical studies.

### Periodontitis induction

Mice were given sulfamethoxazole at 0.87 mg/ml and trimethoprim at 0.17 mg/ml (Hi-Tech Pharmacal Co. Inc., Armityville, NY, USA) in milli-Q water *ad libitum* for 10 days, followed by 3 days without antibiotics. For infection, mice were inoculated with an average 2 × 10^9^ colony-forming units of *P. gingivalis* strain W83 (BAA-308; ATCC, Manassas, VA, USA) in 100 μl phosphate-buffered saline with 2% carboxymethylcellulose (Sigma-Aldrich, St Louis, MO, USA) by oral gavage for 15 days as described previously [28]. The vehicle group received carboxymethylcellulose alone.

### Arthritis induction and evaluation

Mice were immunized with CII as described elsewhere [29]. Briefly, chick CII at 4 mg/ml in 50 mM acetic acid was emulsified in equal volumes of IFA or CFA. IFA was composed of mannide monooleate and heavy paraffin (Fisher Scientific, Hampton, NH, USA). CFA was composed of IFA and freshly ground heat-killed *Mycobacterium tuberculosis* strain H37Ra (BD Biosciences, San Jose, CA, USA). Fifty microliters were injected intradermally at the base of the tail. Arthritis was scored by two calibrated examiners (JTM, EAG) via a visual assessment scoring (VAS) system using a scale of 0 to 4 per limb as described previously [30]. Additionally, paws were measured in the medial–lateral and dorsal–ventral directions by a blinded examiner utilizing a Lange skinfold caliper (Beta Technology Inc., Santa Cruz, CA, USA) at D65, D67, D70, and D72. Micro-computed tomography (micro-CT), histologic scoring, and histomorphometric analysis of the paws were performed.

### *Porphyromonas gingivalis* infection assessment

For *P. gingivalis* colonization determination, the oral microflora was collected at baseline, and at D16, D30, D37, D44, D51, D58, D65, and D73 post inoculation. Bacterial infection was confirmed by polymerase chain reaction of arginine–gingipain (201 base pairs) with minimum detection of 1 × 10^3^ colony-forming units as described previously [10].

### Splenocyte reactivation and cytokine analysis

At D0, D30, D44, D73, spleens were processed and reactivated with 100 μg/ml highly purified lyophilized α1(II) bovine collagen obtained as described previously [31]. Supernatants were collected after 5 days of culture and evaluated for protein expression by Quantibody Mouse TH17 array 1 (RayBiotech, Inc., Norcross, GA, USA).

### Serum analysis

Sera collected at D0, D16, D30, D44 and D73 were evaluated for protein expression by Quantibody Mouse Th17 array 1 (RayBiotech, Inc.). Levels of anti-CII antibodies were evaluated at D44 and D73. Briefly, 96-well plates were coated overnight with 5 μg/ml chick CII, incubated with mouse serum at 1:60, 1:240, and 1:960 dilutions for 1 hour, followed by incubation with alkaline phosphatase-labeled goat anti-mouse IgG1, IgG2a, IgG2b, and IgG3 (SouthernBiotech, Birmingham, AL, USA) at 1:1,000 for 2 hours, and read at 405 nm absorbance.

### Gene expression in gingival tissues, submandibular lymph nodes, and inguinal lymph nodes

Tissues dissected at D0, D30, D44, and D73 were processed for isolation of RNA using the TRIzol method (Invitrogen, Rockville, MD, USA) and purified with the RNeasy mini kit (Qiagen, Valencia, CA, USA). mRNA was reverse transcribed into cDNA using SuperScript II Reverse Transcriptase (Invitrogen). cDNA was then amplified using the TaqMan Universal PCR Master Mix (Applied Biosystems, Carlsbad, CA, USA). The following transcription factors were evaluated: T-bet, GATA-3, RORγt, and Foxp3. Relative quantification of the data generated was carried out using the comparative threshold cycle for quantitative reverse transcription-polymerase chain reaction method.

### Micro-computed tomography analyses

Maxillae were fixed in 10% formalin and then transferred to 70% alcohol. Maxillae were scanned at a resolution of 12 × 12 × 12 μm^3^ voxels using a micro-CT 100 cabinet cone-beam micro-CT system (Scanco USA, Inc., Wayne, PA, USA). Analysis was performed by a calibrated masked examiner (ADT) as previously described with minor modifications [32]. The region of interest encompassed the coronal region of supporting alveolar bone from the mesial edge of the cementum–enamel junction of M1 to the distal edge of the cementum–enamel junction of M2, excluding root tissues. The mean threshold gray-scale value was calculated and used to derive the bone mineral content (mg) and tissue mineral content (mg) using GEHC MicroView Analysis Plus software (GE Healthcare, Little Chalfont, UK). Paws cut above the ankle were placed in 4.5% neutral-buffered zinc-free paraformaldehyde followed by 70% ethanol as described elsewhere [33]. Analysis was performed by a calibrated masked examiner (S-YS) as described previously [34]. The region of interest was defined in digits 2, 3, and 4. Areas of periosteal new bone and cortical bone were discriminated based on the bone resolution of 12 μm^3^ and obtained using the bone analysis command of GEHC MicroView Analysis Plus software (GE Healthcare).

### Histopathological analysis

Maxillae were decalcified in 10% ethylenediamine tetraacetic acid (Acros, Fairlawn, NJ, USA) for 14 days and then embedded in paraffin. Sagittal sections were obtained from each maxilla at the molar region of M1, M2, and M3 and stained with hematoxylin and eosin for descriptive analysis. Paws were decalcified in 14% ethylenediamine tetraacetic acid (Acros) and embedded in paraffin. Transverse paw sections were stained with hematoxylin and eosin, and tartrate-resistant acid phosphatase (TRAP; Sigma-Aldrich) for osteoclast detection. Histopathological scores of joint damage were determined for inflammatory infiltrate, synovitis, cartilage destruction, and bone involvement as described elsewhere [30]. For TRAP staining, slides were deparaffinized in xylene, hydrated in serial ethanol, and incubated in a solution containing diazotized fast garnet, napthol AS-BI phosphate, acetate, and tartrate solution from the Acid Phosphatase, Leukocyte (TRAP) kit (Sigma-Aldrich) as described previously [35]. Osteoclasts were identified as TRAP-positive cells and counted utilizing Osteomeasure software (Osteometrics, Inc., Decatur, GA, USA). Osteoclasts were counted in the phalanges of digits 2, 3 and 4 and expressed as the bone area and bone perimeter. The phalanges were selected by the pathologist involved in the study as a bone region that could be easily traced and quantified and was consistent in all sections.

### Statistical analysis

The study consisted of a total of 15 groups with eight mice per group at four sacrificial time points (D0, D30, D44, D73). Data are expressed as the mean ± standard error of the mean. The differences among groups were statistically assessed by the unpaired two-tailed Student’s *t* test (arthritis development and mRNA expression), one-way analysis of variance (protein expression, antibodies, arthritis scoring, and osteoclast numbers), Dunnet’s multiple comparison test (alveolar bone loss), or linear regression analysis (paw swelling). Comparisons were performed among all groups. Data were analyzed by the GraphPad Prism 5.0 program (GraphPad Software, La Jolla, CA, USA). In all tests, *P* <0.05 was considered statistically significant.

## Results

### Periodontal disease development

**Figure 1:**
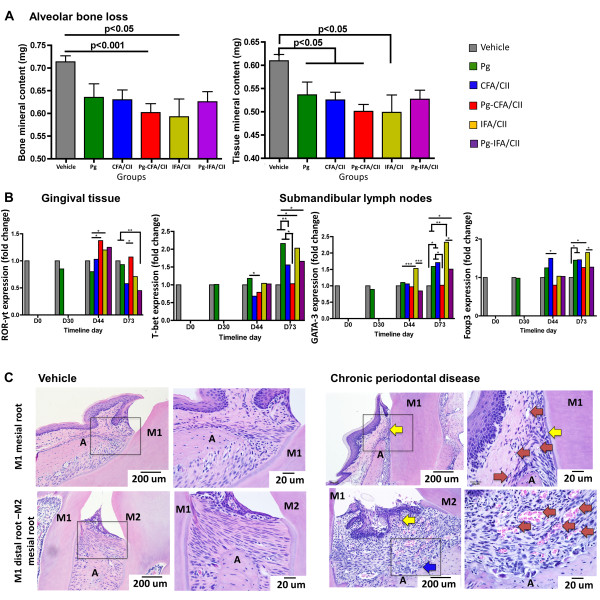
**Periodontal disease development in the periodontitis and arthritis mouse models. (A)** Alveolar bone evaluated at timeline day D73 by micro-computed tomography analysis for bone mineral content and tissue mineral content. Mice that received either *Porphyromonas gingivalis* (Pg), arthritis induction by complete Freund’s adjuvant (CFA)/collagen II (CII), arthritis induction by incomplete Freund’s adjuvant (IFA)/CII, or both *P. gingivalis* and arthritis induction had less alveolar bone compared with vehicle, representing increased bone loss. Data represented as mean ± standard error of the mean, *n* = 8/group, *P* <0.05, Dunn’s multiple comparison test. **(B)** Gingival tissues and submandibular lymph nodes evaluated for mRNA expression showed increased expression of RORγt, T-bet, GATA-3, and Foxp3 in mice that received all treatments (Student’s *t* test, *p≤0.05, **p≤0.01, ***p≤0.001). **(C)** Representative histological sagittal sections of the mesial region of M1 and the interproximal region between M1 and M2 stained with hematoxylin and eosin from mice in the vehicle group and mice that developed chronic periodontal disease. M1 and M2, molars 1 and 2; A, alveolar bone crest; yellow arrows, presence of a dense fibroblast population and disorganized tissue; red arrows, presence of blood vessels; blue arrows, irregular alveolar bone crest, indicating bone resorption.

Confirmation that mice were chronically infected with *P. gingivalis* both at the time of arthritis induction (D30) and during arthritis development (D30 to D65) was achieved via polymerase chain reaction of the oral microflora using arginine–gingipain (Table 1). At D73, *P. gingivalis* colonization was below the level of detection for mice infected with *P. gingivalis* and immunized with CFA/CII and IFA/CII. In comparing the extent of alveolar bone loss, micro-CT analysis demonstrated decreased bone mineral content and tissue mineral content in all treatment groups independent of the presence of *P. gingivalis* when compared with vehicle (Dunnet’s multiple comparison, *P* <0.05 and *P* <0.01) (Figure 1A). Gingival tissue showed increased RORγt expression at D44 in the *P. gingivalis* (Pg)-CFA/CII and IFA/CII groups compared with the Pg group (Figure 1B), confirming that induction of arthritis could induce and influence periodontal disease development. At D44 and D73, a decrease in the submandibular lymph node expression of T-bet was observed if *P. gingivalis*-infected mice were immunized with CFA/CII compared with mice that received *P. gingivalis* alone. The expression of GATA-3 was also altered by *P. gingivalis* oral infection, shown by downregulation in the Pg-IFA/CII group when compared with the IFA/CII group at D44 and D73, and in the Pg-CFA/CII group when compared with the CFA/CII group at D73 (Figure 1B). Mice immunized with IFA/CII had increased GATA-3 expression at D44 and D73 compared with mice in the Pg group. Upregulation of Foxp3 was observed at D73 in mice treated with *P. gingivalis*, CFA/CII, or IFA/CII when compared with vehicle. This datum demonstrates that an active Th cell response was developing in the oral-related tissues by both CII immunizations and by oral infection with *P. gingivalis*. Finally, histological sections confirmed chronic periodontitis by the presence of enlarged blood vessels, irregular alveolar bone crest, tissue remodeling and dense fibroblast populations above the crestal bone of the mesial M1 region as well as the distal M1 and mesial M2 region in mice from all treatment groups (Figure 1C). These features were not present in mice from the vehicle group, and interestingly no major additive effect of treatment combinations (*P. gingivalis* with CII immunizations) could be observed. In summary, immunization with CII or infection with *P. gingivalis* induced the development of chronic PD in the mice with corresponding development of Th cell responses.

**Table 1:** ***Porphyromonas gingivalis *** **oral colonization during periodontitis and arthritis development**

**Group**	**Day 0**	**Day 16**	**Day 23**	**Day 30**	**Day 37**	**Day 44**	**Day 51**	**Day 58**	**Day 65**	**Day 73**
Baseline	0/8									
D30 Vehicle	0/8			0/8						
D30 Pg	0/8	6/8	6/8	4/8						
D44 Vehicle	0/8			0/8						
D44 Pg	0/8	8/8	8/8	8/8	8/8	8/8				
D44 CFA	0/8					0/8				
D44 Pg-CFA	0/8	8/8	8/8	8/8	8/8	8/8				
D44 IFA	0/8					0/8				
D44 Pg-IFA	0/8	8/8	8/8	8/8	8/8	8/8				
D73 Vehicle	0/8									0/8
D73 Pg	0/8	8/8	8/8	8/8	7/8	8/8	8/8	8/8	6/8	8/8
D73 CFA	0/8									0/8
D73 Pg-CFA	0/8	6/8	7/8	8/8	8/8	8/8	6/8	8/8	8/8	0/8
D73 IFA	0/8									0/8
D73 Pg-IFA	0/8	4/8	8/8	8/8	5/8	8/8	7/8	2/8	6/8	0/8

Murine oral microflora collected at timeline days D0, D16, D23, D30, D37, D44, D51, D58, D65, and D73 were evaluated by polymerase chain reaction of arginine–gingipain (201 base pairs). Values represent the number of infected mice/number of total mice in each group (*n* = 8). Mice that were not infected with *P. gingivalis* were not evaluated weekly, but only at baseline and sacrifice. CFA, complete Freund’s adjuvant; IFA, incomplete Freund’s adjuvant; Pg, *P. gingivalis*.

### Arthritis progression is increased by *P. gingivalis* oral infection in mice challenged for arthritis with CFA/CII

**Figure 2:**
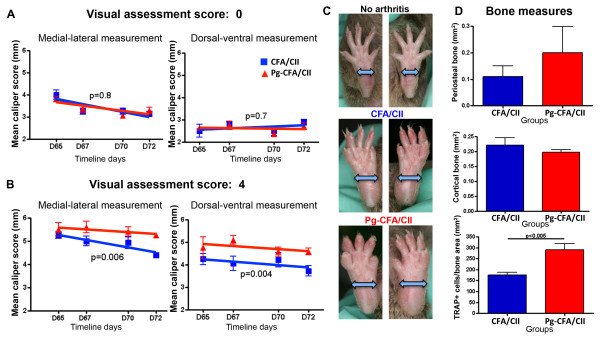
**Increased swelling and osteoclast numbers in mice with *Porphyromonas gingivalis *oral infection.** Paws measured in the medial–lateral and dorsal–ventral dimensions at timeline days D65, D67, D70, and D72 (35, 37, 40, and 42 days after complete Freund’s adjuvant (CFA)/collagen II (CII) immunization) were compared with **(A)** paws with visual assessment score of 0 (no arthritis) and **(B)** paws that developed a visual assessment score of 4. **(C)** Representative figures of hind paws that developed score 0 (upper panel), score 4 with medial–lateral measurement of 4 mm in mice from the CFA/CII group (middle panel), and score 4 with medial–lateral measurements of 5.0 mm and 5.5 mm in mice from the *P. gingivalis* (Pg)-CFA/CII group (lower panel). Error bars represent ± standard error of the mean (SEM) of four to six mice per group, *P* <0.05, linear regression analysis. **(D)** No differences between groups were observed in paws evaluated by micro-computed tomography for periosteal new bone and cortical bone destruction bone area; however, the osteoclast number by tartrate-resistant acid phosphatase (TRAP)-positive staining was significantly increased in mice with oral *P. gingivalis*. Error bars represent ± SEM of four to six mice per group, *P* <0.05, Student’s *t* test.

VAS showed no significant differences in the final arthritis incidence between mice in the Pg-CFA/CII and CFA/CII groups (7/8 and 8/8 respectively). Interestingly, changes in the severity of arthritis were induced by *P. gingivalis* after arthritis had developed in the entire paw (VAS 4). Paw swelling was significantly increased in both the medial–lateral and dorsal–ventral dimensions once arthritis developed in the entire hind paw in mice orally infected with *P. gingivalis* and further immunized with CFA/CII (Figure 2A,B,C). The same pattern was observed in the front paws (data not shown). Paws with VAS of 4 were further analyzed for bone loss by micro-CT and the presence of active osteoclasts by TRAP staining. While our results did not show significant differences in amount of bone resorption by micro-CT analysis, mice in the Pg-CFA/CII group had an increased number of osteoclasts/bone area when compared with the CFA/CII group alone (Figure 2D). These findings demonstrate that even with a robust model for arthritis induction utilizing adjuvant that contains *M. tuberculosis* fragments within CFA/CII in the process of immunization, *P. gingivalis* chronic oral infection altered the progression of arthritis by increasing paw swelling and osteoclast recruitment.

### Arthritis development is altered by *P. gingivalis* oral infection in mice induced for arthritis with IFA/CII

**Figure 3:**
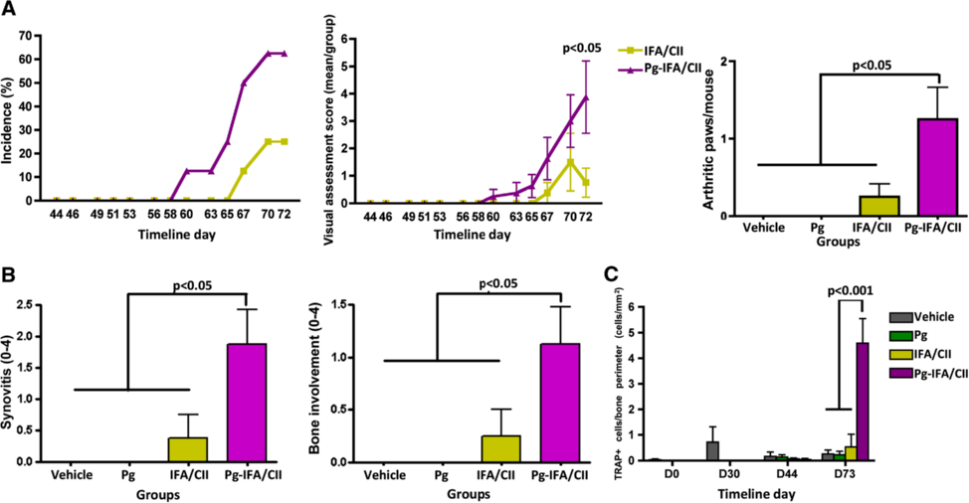
***Porphyromonas gingivalis *oral infection increased arthritis development in mice immunized with collagen II in incomplete Freund’s adjuvant.** Mice infected with *P. gingivalis* (Pg) in the oral cavity for 15 days were immunized with incomplete Freund’s adjuvant (IFA)/collagen II (CII) 15 days later as described in Methods. **(A)** Arthritis development and severity was scored by a visual assessment score on a 0 to 4 scale. The total number of arthritic paws was significantly higher in mice with *P. gingivalis* oral infection and IFA/CII immunization compared with IFA/CII alone. **(B)** Paws collected at D73 demonstrated increased synovitis and greater bone involvement if mice had *P. gingivalis* oral infection and IFA/CII immunization compared with IFA/CII alone. **(C)** Paws collected at timeline D0, D30, D44, and D73 were scored for tartrate-resistant acid phosphatase (TRAP)-positive cells. Increased osteoclasts numbers were observed at D73 if mice had *P. gingivalis* infection and IFA/CII immunization. Error bars represent ± standard error of the mean of eight mice per group, *P* <0.05, Student’s *t* test and analysis of variance.

Increased incidence and severity were observed in mice from the Pg-IFA/CII group compared with the IFA/CII group alone (D58 in the Pg-IFA/CII group and D65 in the IFA/CII group, Figure 3A). In addition, the final mean VAS and mean number of arthritic paws/group was significantly higher at D73 in mice from the Pg-IFA/CII group compared with the IFA/CII group (Figure 3A). Histological scoring of the paws confirmed the clinical findings, demonstrating that mice gavaged with *P. gingivalis* and immunized with IFA/CII displayed increased synovial thickening and pannus formation as well as bony erosions when compared with IFA/CII alone (Figure 3B). Paws evaluated by TRAP staining showed that mice infected with *P. gingivalis* followed by IFA/CII immunization had an increased number of osteoclasts/bone perimeter (Figure 3C). In sum, these results show that a chronic oral *P. gingivalis* infection initiated prior to IFA/CII immunization increased the incidence and severity of CIA and was associated with increased osteoclast numbers.

### *Porphyromonas gingivalis* increased serum Th17 responses

**Figure 4:**
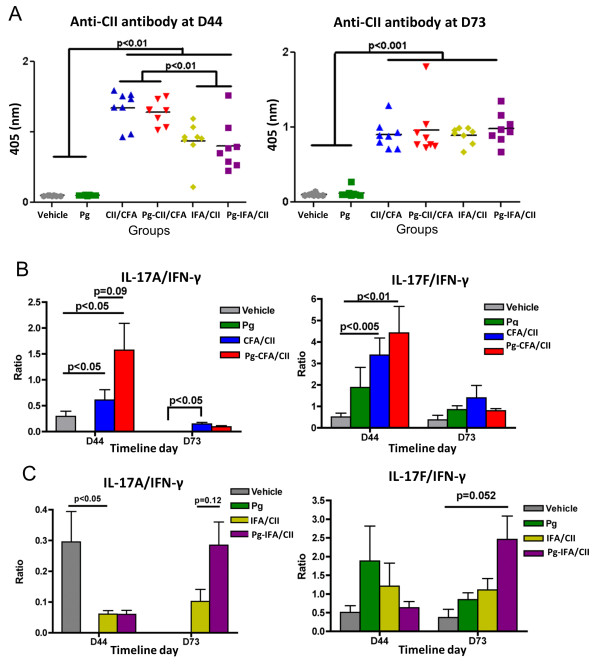
**Effect of *Porphyromonas gingivalis *on serum responses after collagen-induced arthritis induction.** Mice gavaged with *P. gingivalis* (Pg) in the oral cavity for 15 days (D0 to D15) were immunized with collagen II (CII) emulsified in complete Freund’s adjuvant (CFA) and incomplete Freund’s adjuvant (IFA) 15 days later (D30), as described in Methods. **(A)** Antibodies to CII were measured by enzyme-linked immunosorbent assay at D44 and at D73. Serum collected at D44 and D73 were evaluated for interleukin (IL)-17A/interferon (IFN)γ and IL-17 F/IFNγ ratios in **(B)** CFA/CII immunizations and **(C)** IFA/CII immunizations. Error bars represent ± standard error of the mean of eight mice per group, *P* <0.05, analysis of variance and Student’s *t* test. *P* <0.2 results are also presented.

It was anticipated that the immunological development of arthritis in IFA/CII groups might be slower than in CFA/CII groups [36]. Indeed, our results show that mice immunized with IFA/CII had a significantly lower anti-CII antibody response compared with mice immunized with CFA/CII at D44, but reached similar values at D73, independent of oral infection with *P. gingivalis* (Figure 4A). This demonstrates that *P. gingivalis* did not have an effect on the antibody response to CII. Rather than the absolute value of an individual cytokine, the balance of serum Th17 and Th1 responses was previously shown to be associated with the degree of joint inflammation, with increased IL-17A/IFNγ ratios present in mice that developed more severe CIA [30]. Interestingly, the balance of serum IL-17A/IFNγ showed a trend to be increased (*P* = 0.09) at D44 if *P. gingivalis* oral infection was present in mice developing arthritis in the CFA/CII group compared with CFA/CII alone, and a nonsignificant trend in IL-17F/IFNγ ratios (Figure 4B). An increased trend in IL-17F/IFNγ (P = 0.055) was found at D73 if mice had *P. gingivalis* oral infection and IFA/CII immunization when compared with mice in the vehicle group. Our results show that *P. gingivalis* oral infection favored a Th17 systemic response by increasing IL-17 levels and decreasing IFNγ serum levels.

### *Porphyromonas gingivalis* increased Th17 responses in collagen II-reactivated splenocytes

**Figure 5:**
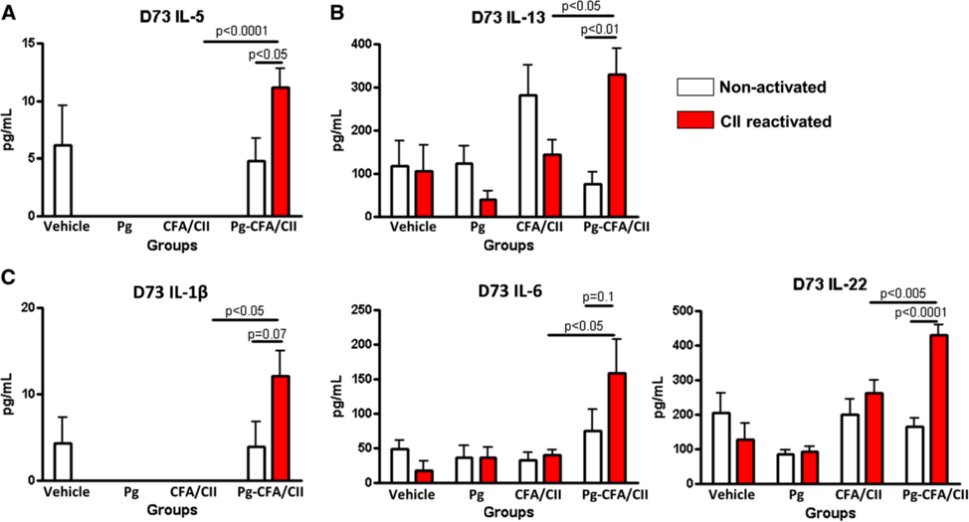
**Mice exposed to *Porphyromonas gingivalis *prior to immunization with collagen II and complete Freund’s adjuvant exhibit increased splenic T-helper type 17-related responses.** Murine splenocytes were isolated from mice that received 15 oral gavages of *P. gingivalis* (Pg) followed by complete Freund’s adjuvant (CFA)/collagen II (CII) immunization at day D30 according to Methods. Splenocytes collected at D73 were reactivated *in vitro* with CII. Supernatants evaluated for protein expression demonstrated increased expression of **(A)** T-helper type (Th)1-related cytokine interleukin (IL)-5, **(B)** Th2-related cytokine IL-13, and **(C)** Th17-related cytokines IL-1β, IL-6, and IL-22 if mice had *P. gingivalis* infection in addition to CFA/CII immunization. Error bars represent ± standard error of the mean of eight mice per group, *P* <0.05, analysis of variance followed by Tukey’s multiple comparison test, and Student’s *t* test.

**Figure 6:**
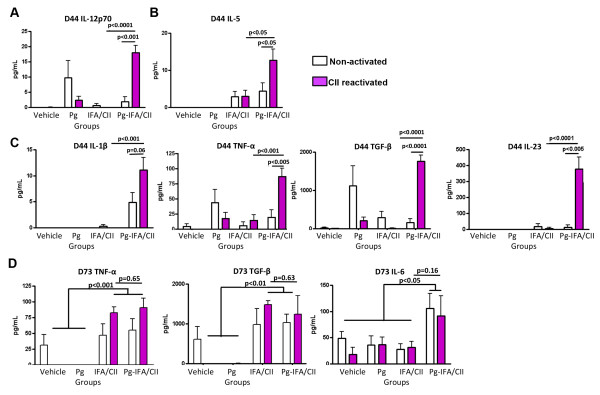
**Mice exposed to *Porphyromonas gingivalis *prior to immunization with collagen II and incomplete Freund’s adjuvant generates increased splenic T-helper type 17-related responses.** Murine splenocytes were isolated from mice that received 15 oral gavages of *P. gingivalis* (Pg) followed by incomplete Freund’s adjuvant (IFA)/collagen II (CII) immunization at day D30 according to Methods. Splenocytes were collected at D44 and D73 and reactivated *in vitro* with CII. Supernatants collected and evaluated for protein expression at D44 demonstrated increased **(A)** T-helper type (Th)1-related response via interleukin (IL)-12p70, **(B)** increased Th2-related response via IL-5, and **(C)** increased Th17-related responses via IL-1β, tumor necrosis factor (TNF)-α, transforming growth factor beta (TGF-β), and IL-23 if mice had *P. gingivalis* infection in mice immunized with IFA/CII. **(D)** At D73, immunized mice demonstrated increased expression of TNFα and TGF-β, with IL-6 being significantly upregulated independent of *in vitro* reactivation. Error bars represent ± standard error of the mean of eight mice per group, *P* <0.05, analysis of variance followed by Tukey’s multiple comparison test, and Student’s *t* test.

Supernatants from murine splenocytes treated with CII were evaluated for protein expression. Mice with oral *P. gingivalis* infection demonstrated significantly increased levels of Th1-related, Th2-related and Th17-related cytokines once mice were immunized with either CFA/CII or IFA/CII when compared with mice that had no *P. gingivalis* infection. Mice in the Pg-CFA/CII group had significant increased expression of Th2-related cytokines IL-5 and IL-13 (Figure 5A,B), as well as Th17-related cytokines IL-1β, IL-6, and IL-22 when compared with CFA/CII alone at D73 (Figure 5C). The majority of changes induced by *P. gingivalis* oral infection were observed on D44 (Figure 6A,B,C), and less were observed on D73 (Figure 6D). At D44, mice in the Pg-IFA/CII group had significantly increased levels of Th1-related cytokine IL-12p70 (Figure 6A), Th2-related cytokine IL-5 (Figure 6B), and Th17-related cytokines IL-1β, TNFα, TGF-β, and IL-23 (Figure 6C) when compared with IFA/CII alone. At D73, Th17-related cytokines TGF-β and TNFα were significantly upregulated in CII immunized mice independent of the oral *P. gingivalis* infection, while IL-6 levels were significantly upregulated independent of CII reactivation if mice were infected with *P. gingivalis* (Figure 6D). mRNA isolated from inguinal lymph nodes demonstrated increased expression of T-bet in both the Pg*-*CFA/CII and CFA/CII groups compared with vehicle at D44 (data not shown). In summary, our results show the expression of factors involved in the development of Th1, Th2, and Th17 cells, but the majority of the differences between mice that received oral *P. gingivalis* infection followed by CII immunization compared with CII immunization alone were observed in Th17-related cytokines. Although some cytokines can be expressed by T cells and B cells in the mixed splenic population we evaluated *in vitro*, the majority of cytokines are expressed (not exclusively) by monocytes/macrophages and dendritic cells, including IL-1β, IL-6, IL-22, IL-12p70, TNFα, IL-6, and IL-23. These results suggest that the oral *P. gingivalis* infection initiated prior to arthritis induction sensitized innate immune cells and increased cytokine responses favoring Th17 cells, which ultimately led to increased arthritis development and progression.

## Discussion

RA is a chronic inflammatory disease clinically associated with PD [14]. Some studies demonstrate that patients with RA demonstrate clinical and serological improvements if periodontal therapy is provided [23-25], suggesting that a chronic oral infection can alter established RA. Here we show for the first time that a chronic oral infection with bacterium *P. gingivalis* favored Th17-driven responses that ultimately influenced CIA development and progression.

Both CIA and PD are inflammatory, Th cell-mediated diseases [7,30]. Cytokine modulation therapies, such as anti-TNFα, anti-IL-23p19 and anti-IL22, are shown to alter disease development in preclinical and/or clinical settings [16-19]. Interestingly, other infections have been demonstrated to affect proinflammatory cytokines and CIA development [37,38]. Helminth product ES-62 can alter the Th17 network at multiple sites and ultimately protects mice from developing CIA [39]. Understanding how chronic periodontitis can modulate the cytokine network driving arthritic immune responses before clinical bone destruction takes place is therefore of great interest in relation to developing preventive periodontal therapies in susceptible populations.

Several immunological processes need to occur for arthritis to develop, including activation of antigen-presenting cells by pattern-recognition receptors, T-cell and B-cell polarization, and finally osteoclast activation. Arthritis induction with CII in combination with either CFA or seldom-used IFA allowed identification of the immunological phase of arthritis development most pronouncedly affected by *P. gingivalis*. The significantly lower arthritis incidence and severity and higher day of onset of arthritis in mice immunized with CII and IFA has led to the use of CII and CFA for arthritis induction in the great majority of the studies [36,40]. The effect of *P. gingivalis* in CIA development was observed in mice immunized with either CFA/CII or IFA/CII (Figures 2 and 3). However, the diminished activation of the innate immune response, including antigen-presenting cells, by the absence of *M. tuberculosis* in IFA/CII brought out increased effects of *P. gingivalis* in CIA development. This observation suggests that the majority of effects induced by *P. gingivalis* were during the innate immune response. Our *in vitro* results strengthen this finding by showing that the majority of cytokines upregulated by *P. gingivalis* were derived from monocytes/macrophages and dendritic cells (Figures 5 and 6). The results therefore indicate that the most pronounced effects induced by *P. gingivalis* in CIA were by sensitizing innate immune cells favoring a Th17 response.

It was previously shown that the balance of Th1, Th2, and Th17 responses could control the immune events leading to bone destruction. Our data showed a trend towards increased Th17/Th1 ratios in mice with oral *P. gingivalis* induced for arthritis with either CFA/CII or IFA/CII (Figure 4B,C), which was correlated with increased paw swelling, accelerated development of CIA, and increased osteoclast numbers (Figures 2 and 3). In accordance with our results, a higher Th17/Th1 ratio has been observed in mice that develop more severe arthritis [30]. The cytokine network alteration induced by *P gingivalis* in the serum was further validated by a spleen cell *in vitro* assay. Increased expression of Th2-related cytokines IL-5 and IL-13 and Th17-related cytokines IL-1β, IL-6, and IL-22 at D73 were found in mice infected with *P gingivalis* prior to CFA/CII immunizations (Figure 5)*.* IL-5 and IL-13 are cytokines related to arthritis suppression [41,42], while tissue-destructive cytokines IL-1β and IL-6 induce Th17 cells and promote osteoclastogenesis [43]. IL-22, which is significantly upregulated in the Pg-CFA/CII group (Figure 5E), can be expressed by Th17 cells and assists in promoting inflammation [44]. In the IFA/CII groups, the levels of IL-5 and IL-12 (Figure 5A,B) and of Th17-related cytokines IL-1β, TNFα, TGF-β, and IL-23 were significantly higher at D44 if mice had *P. gingivalis* infection (Figure 6C). Interestingly, inflammatory cytokines such as TNFα and IL-1β are known to potentiate the effects of IL-17 and IL-17 F [45]. IL-23 is known to be one essential factor required for the expansion of pathogenic Th17 cells and development of autoimmunity [46]. At D73, increased expression of IL-6 was observed in mice immunized with *P. gingivalis*, indicating that a Th17 response was in the process of development. TGF-β, which was present at D73 independent of *P. gingivalis* infection, is known to promote differentiation of cells into Th17 cells in the presence of IL-6 [43], which was being constitutively expressed by splenocytes independent of CII reactivation in the Pg-IFA/CII group (Figure 6D). Th17-related cytokines promote osteoclastogenesis either directly by inducing RANKL or indirectly by inducing IL-17 [16,43]. Because mice infected with *P. gingivalis* showed increased arthritis progression, development and osteoclast numbers, we conclude that the net effect of Th17 destructive response was stronger than the protection that could be provided by Th1-related and Th2-related cytokines.

The timing of cytokine modulation during the immunological events that take place once CIA is induced is shown to be an important factor to consider when modifying the final arthritis development. For example, neutralizing IL-23 before clinical signs of arthritis take place can suppress CIA severity, while anti-IL-23p19 antibody treatment after the first signs of CIA does not affect the disease development [18]. In accordance with our results, it has been previously shown that prior *P. gingivalis* oral infection increased arthritis development in the collagen-antibody induced arthritis model [27]. Interestingly, *P. gingivalis* infection initiating after arthritis induction was shown to have no effects on arthritis development in the pristane-induced model of arthritis [47]. Future studies with different time frames between disease inductions will assist in clarifying whether the influence of *P. gingivalis* in arthritis development is dependent on timing.

The oral gavage model with *P. gingivalis* is known as a classical method for inducing periodontitis in susceptible rodents and can be utilized for studying the association of periodontal disease with other systemic conditions [48]. While the effect of arthritis induction on periodontitis development was not the focus of this study, we observed alveolar bone loss in mice that received either CII immunizations or *P. gingivalis* oral infection, which was further supported by increased local expression of several transcription factors (Figure 1). The development of periodontitis during arthritis induction is in agreement with several murine studies, including in the CIA model [49], the adjuvant arthritis model [50], the chronic-antigen induced arthritis model [51], and the pristane-induced arthritis model [47]. Periodontitis development in the CIA model was characterized by increased osteoclast activity and adipocyte production, and decreased osteoblastic activity in alveolar bone cells. These results indicate that the local alveolar cells develop an altered behavior with immunizations that leads to alveolar bone destruction. However, the Th cell responses and oral microflora in these mice were not evaluated. In our study, we showed that immunized mice did not have an increase in *P. gingivalis* colonization (Table 1), ruling out the possibility that alveolar bone loss in the CFA/CII and IFA/CII groups was directly due to oral *P. gingivalis* infection. Recently, a comprehensive analysis of the subgingival microbiota of patients with new-onset and chronic RA demonstrated that bacterium *Anaeroglobus geminatus* correlated with the presence of anti-citrullinated protein antibodies and rheumatoid factor, while *Prevotella* and *Leptotrichia* species were the only taxa observed in patients with new-onset RA irrespective of PD status [52]. This implies that the oral microflora shifts may be distinct in the RA population and that additional periodontal bacteria should be explored in the context of arthritis. Interestingly, control of the oral microflora with antimicrobial treatment stopped alveolar bone loss in both the pristine model of arthritis [47] and the chronic antigen-induced arthritis models [51]. Although no further characterization of the microflora was performed in that study, this observation suggests that a microbial shift occurs in mice induced for arthritis. Interestingly, our analysis showed that mice induced for arthritis had a more sporadic oral infection with *P. gingivalis* compared with mice with no arthritis induction (Table 1). This observation indicates that the oral microflora of immunized mice was interfering with *P. gingivalis* colonization and strengthens the hypothesis that a microbial shift occurs in immunized mice. Increasing the understanding of the oral microflora and potential microbiota shift that may occur with arthritis induction and development is of interest. Exploration of the effect of additional periodontal bacteria in arthritis development is of interest. Still, RA patients with severe periodontitis show a more robust antibody response against *P. gingivalis* than non-RA controls [53], suggesting that infection with this bacterium in RA patients could be important.

## Conclusion

Our data indicate that alveolar bone loss occurs in the presence of oral *P. gingivalis* or CII immunization. Oral infection of mice with *P. gingivalis* altered the immune development of arthritis primarily via Th17-driven responses, with increased paw swelling, accelerated development, higher incidence of arthritis, and increased paw osteoclast numbers. Our data also suggest that the more pronounced effects observed were related to an augmentation of the innate immune response. These results are important in furthering our understanding for the potential of an oral chronic infection in altering arthritis condition in susceptible patients, and may have important implications for developing future preventive strategies.

## Abbreviations

CFA: Complete Freund’s adjuvant; CIA: Collagen-induced arthritis; CII: Collagen II; Foxp3: Transcription factor specific for T-regulatory cells; GATA-3: Transcription factor specific for T-helper type 2 cells; IFA: Incomplete Freund’s adjuvant; IFN: Interferon; IL: Interleukin; micro-CT: Micro-computed tomography; PD: Periodontal disease; Pg: *Porphyromonas gingivalis*; RA: Rheumatoid arthritis; RORγt: Transcription factor specific for T-helper type 17 cells; T-bet: Transcription factor specific for T-helper type 1 cells; TGF-β: Transforming growth factor beta; Th: T helper type; TNF: Tumor necrosis factor; TRAP: Tartrate-resistant acid phosphatase; VAS: Visual assessment scoring.

## Competing interests

The authors declare that they have no competing interests.

## Authors’ contributions

JTM drafted the manuscript, performed the study design, performed the experiments, contributed with reagents/materials/data acquisition, performed analysis and interpretation of data, and performed statistical analysis. EAG performed the experiments, contributed with data acquisition, and performed analysis and interpretation of data, and statistical analysis. RS contributed with materials/data acquisition, and performed analysis and interpretation of data, and statistical analysis. ADT contributed with reagents/materials/data acquisition, and performed analysis and interpretation of data. S-YS contributed with reagents/materials/data acquisition, and performed analysis and interpretation of data. JS contributed with reagents/materials/data acquisition. DB contributed with reagents/materials/data acquisition. AB contributed with reagents/materials/data acquisition, and performed analysis and interpretation of data, and statistical analysis. JJ performed analysis and interpretation of data. SKL contributed with reagents/materials/data acquisition, and performed analysis and interpretation of data. GN performed the study design, contributed with reagents/materials/data acquisition, and performed analysis and interpretation of data. DAF performed the study design, contributed with reagents/materials/data acquisition, and performed analysis and interpretation of data. WVG performed the study design, contributed with reagents/materials/data acquisition, and performed analysis and interpretation of data. All authors contributed and approved the final version to be published.

## References

[B1] Albandar JM Underestimation of periodontitis in NHANES surveys J Periodontol 2011 15 337 341 10.1902/jop.2011.100638 21214340

[B2] Hajishengallis G Darveau RP Curtis MA The keystone-pathogen hypothesis Nat Rev Microbiol 2012 15 717 725 10.1038/nrmicro2873 22941505 PMC3498498

[B3] Socransky SS Smith C Haffajee AD Subgingival microbial profiles in refractory periodontal disease J Clin Periodontol 2002 15 260 268 10.1034/j.1600-051x.2002.290313.x 11940147

[B4] Burns E Bachrach G Shapira L Nussbaum G Cutting Edge: TLR2 is required for the innate response to Porphyromonas gingivalis: activation leads to bacterial persistence and TLR2 deficiency attenuates induced alveolar bone resorption J Immunol 2006 15 8296 8300 17142724 10.4049/jimmunol.177.12.8296

[B5] Gaddis DE Michalek SM Katz J TLR4 signaling via MyD88 and TRIF differentially shape the CD4^+^ T cell response to Porphyromonas gingivalis hemagglutinin B J Immunol 2011 15 5772 5783 10.4049/jimmunol.1003192 21498664 PMC3809913

[B6] Chung WO An JY Yin L Hacker BM Rohani MG Dommisch H DiJulio DH Interplay of protease-activated receptors and NOD pattern recognition receptors in epithelial innate immune responses to bacteria Immunol Lett 2010 15 113 119 10.1016/j.imlet.2010.02.006 20219537 PMC2885501

[B7] Garlet GP Destructive and protective roles of cytokines in periodontitis: a re-appraisal from host defense and tissue destruction viewpoints J Dent Res 2010 15 1349 1363 10.1177/0022034510376402 20739705

[B8] Yu JJ Ruddy MJ Conti HR Boonanantanasarn K Gaffen SL The interleukin-17 receptor plays a gender-dependent role in host protection against Porphyromonas gingivalis-induced periodontal bone loss Infect Immun 2008 15 4206 4213 10.1128/IAI.01209-07 18591228 PMC2519446

[B9] Eskan MA Jotwani R Abe T Chmelar J Lim JH Liang S Ciero PA Krauss JL Li F Rauner M Hofbauer LC Choi EY Chung KJ Hashim A Curtis MA Chavakis T Hajishengallis G The leukocyte integrin antagonist Del-1 inhibits IL-17-mediated inflammatory bone loss Nat Immunol 2012 15 465 473 10.1038/ni.2260 22447028 PMC3330141

[B10] Marchesan JT Morelli T Lundy SK Jiao Y Lim S Inohara N Nunez G Fox DA Giannobile WV Divergence of the systemic immune response following oral infection with distinct strains of Porphyromonas gingivalis Mol Oral Microbiol 2012 15 483 495 10.1111/omi.12001 23134613 PMC4106254

[B11] Miyauchi S Maekawa T Aoki Y Miyazawa H Tabeta K Nakajima T Yamazaki K Oral infection with Porphyromonas gingivalis and systemic cytokine profile in C57BL/6.KOR-ApoE shl mice J Periodontal Res 2012 15 402 408 10.1111/j.1600-0765.2011.01441.x 22097957

[B12] Friedewald VE Kornman KS Beck JD Genco R Goldfine A Libby P Offenbacher S Ridker PM Van Dyke TE Roberts WC The American Journal of Cardiology and Journal of Periodontology Editors’ Consensus: periodontitis and atherosclerotic cardiovascular disease Am J Cardiol 2009 15 59 68 10.1016/j.amjcard.2009.05.002 19576322

[B13] Lamster IB Lalla E Borgnakke WS Taylor GW The relationship between oral health and diabetes mellitus J Am Dent Assoc 2008 15 19S 24S 18809650 10.14219/jada.archive.2008.0363

[B14] de Pablo P Dietrich T McAlindon TE Association of periodontal disease and tooth loss with rheumatoid arthritis in the US population J Rheumatol 2008 15 70 76 18050377

[B15] Brooks PM The burden of musculoskeletal disease – a global perspective Clin Rheumatol 2006 15 778 781 10.1007/s10067-006-0240-3 16609823

[B16] McInnes IB Schett G Cytokines in the pathogenesis of rheumatoid arthritis Nat Rev Immunol 2007 15 429 442 10.1038/nri2094 17525752

[B17] Mirrielees J Crofford LJ Lin Y Kryscio RJ Dawson DR 3rd Ebersole JL Miller CS Rheumatoid arthritis and salivary biomarkers of periodontal disease J Clin Periodontol 2010 15 1068 1074 10.1111/j.1600-051X.2010.01625.x 20880053 PMC2980566

[B18] Cornelissen F Asmawidjaja PS Mus AM Corneth O Kikly K Lubberts E IL-23 dependent and independent stages of experimental arthritis: no clinical effect of therapeutic IL-23p19 inhibition in collagen-induced arthritis PLoS One 2013 15 e57553 10.1371/journal.pone.0057553 23469022 PMC3585376

[B19] Marijnissen RJ Koenders MI Smeets RL Stappers MH Nickerson-Nutter C Joosten LA Boots AM van den Berg WB Increased expression of interleukin-22 by synovial Th17 cells during late stages of murine experimental arthritis is controlled by interleukin-1 and enhances bone degradation Arthritis Rheum 2011 15 2939 2948 10.1002/art.30469 21618207

[B20] Sarkar S Zhou X Justa S Bommireddy SR Interleukin-22 reduces the severity of collagen-induced arthritis in association with increased levels of interleukin-10 Arthritis Rheum 2013 15 960 971 10.1002/art.37849 23334981 PMC3618496

[B21] Chen HH Huang N Chen YM Chen TJ Chou P Lee YL Chou YJ Lan JL Lai KL Lin CH Chen DY Association between a history of periodontitis and the risk of rheumatoid arthritis: a nationwide, population-based, case–control study Ann Rheum Dis 2012 15 1206 1211 22941768 10.1136/annrheumdis-2012-201593

[B22] Mikuls TR Thiele GM Deane KD Payne JB O’Dell JR Yu F Sayles H Weisman MH Gregersen PK Buckner JH Keating RM Derber LA Robinson WH Holers VM Norris JM Porphyromonas gingivalis and disease-related autoantibodies in individuals at increased risk of rheumatoid arthritis Arthritis Rheum 2012 15 3522 3530 10.1002/art.34595 22736291 PMC3467347

[B23] Erciyas K Sezer U Ustun K Pehlivan Y Kisacik B Senyurt S Tarakcioglu M Onat A Effects of periodontal therapy on disease activity and systemic inflammation in rheumatoid arthritis patients Oral Dis 2012 15 394 400 22998534 10.1111/odi.12017

[B24] Ortiz P Bissada NF Palomo L Han YW Al-Zahrani MS Panneerselvam A Askari A Periodontal therapy reduces the severity of active rheumatoid arthritis in patients treated with or without tumor necrosis factor inhibitors J Periodontol 2009 15 535 540 10.1902/jop.2009.080447 19335072 PMC2884010

[B25] Al-Katma MK Bissada NF Bordeaux JM Sue J Askari AD Control of periodontal infection reduces the severity of active rheumatoid arthritis J Clin Rheumatol 2007 15 134 137 10.1097/RHU.0b013e3180690616 17551378

[B26] Ogrendik M Rheumatoid arthritis is linked to oral bacteria: etiological association Mod Rheumatol 2009 15 453 456 10.1007/s10165-009-0194-9 19554393

[B27] Cantley MD Haynes DR Marino V Bartold PM Pre-existing periodontitis exacerbates experimental arthritis in a mouse model J Clin Periodontol 2011 15 532 541 10.1111/j.1600-051X.2011.01714.x 21434962

[B28] Kobayashi R Kono T Bolerjack BA Fukuyama Y Gilbert RS Fujihashi K Ruby J Kataoka K Wada M Yamamoto M Fujihashi K Induction of IL-10-producing CD4^+^ T-cells in chronic periodontitis J Dent Res 2011 15 653 658 10.1177/0022034510397838 21335536 PMC3144111

[B29] Brand DD Latham KA Rosloniec EF Collagen-induced arthritis Nat Protoc 2007 15 1269 1275 10.1038/nprot.2007.173 17546023

[B30] Sarkar S Cooney LA White P Dunlop DB Endres J Jorns JM Wasco MJ Fox DA Regulation of pathogenic IL-17 responses in collagen-induced arthritis: roles of endogenous interferon-gamma and IL-4 Arthritis Res Ther 2009 15 R158 10.1186/ar2838 19852819 PMC2787258

[B31] Rosloniec EF Cremer M Kang AH Myers LK Brand DD Collagen-induced arthritis Curr Protoc Immunol 2010 15 Unit 15.5.1–25 10.1002/0471142735.im1505s89 20376842

[B32] Park CH Abramson ZR Taba M Jr Jin Q Chang J Kreider JM Goldstein SA Giannobile WV Three-dimensional micro-computed tomographic imaging of alveolar bone in experimental bone loss or repair J Periodontol 2007 15 273 281 10.1902/jop.2007.060252 17274716 PMC2581750

[B33] Schett G Tuerk B Bone histomorphometry in arthritis models Methods Mol Med 2007 15 269 283 10.1007/978-1-59745-401-8_17 17951665

[B34] Barck KH Lee WP Diehl LJ Ross J Gribling P Zhang Y Nguyen K van Bruggen N Hurst S Carano RA Quantification of cortical bone loss and repair for therapeutic evaluation in collagen-induced arthritis, by micro-computed tomography and automated image analysis Arthritis Rheum 2004 15 3377 3386 10.1002/art.20557 15476252

[B35] Boabaid F Berry JE Koh AJ Somerman MJ McCcauley LK The role of parathyroid hormone-related protein in the regulation of osteoclastogenesis by cementoblasts J Periodontol 2004 15 1247 1254 10.1902/jop.2004.75.9.1247 15515341

[B36] Matthys P Vermeire K Mitera T Heremans H Huang S Schols D De Wolf-Peeters C Billiau A Enhanced autoimmune arthritis in IFN-gamma receptor-deficient mice is conditioned by mycobacteria in Freund’s adjuvant and by increased expansion of Mac-1+ myeloid cells J Immunol 1999 15 3503 3510 10477624

[B37] Song X Shen J Wen H Zhong Z Luo Q Chu D Qi Y Xu Y Wei W Impact of Schistosoma japonicum infection on collagen-induced arthritis in DBA/1 mice: a murine model of human rheumatoid arthritis PLoS One 2011 15 e23453 10.1371/journal.pone.0023453 21858123 PMC3152573

[B38] Taurog JD Leary SL Cremer MA Mahowald ML Sandberg GP Manning PJ Infection with Mycoplasma pulmonis modulates adjuvant- and collagen-induced arthritis in Lewis rats Arthritis Rheum 1984 15 943 946 10.1002/art.1780270816 6331830

[B39] Pineda MA McGrath MA Smith PC Al-Riyami L Rzepecka J Gracie JA Harnett W Harnett MM The parasitic helminth product ES-62 suppresses pathogenesis in collagen-induced arthritis by targeting the interleukin-17-producing cellular network at multiple sites Arthritis Rheum 2012 15 3168 3178 10.1002/art.34581 22729944

[B40] Ellis JS Chain BM Cooke A Ibrahim MA Katz DR Adjuvant composition determines the induction of type II collagen-induced arthritis Scand J Immunol 1992 15 49 56 10.1111/j.1365-3083.1992.tb02939.x 1615283

[B41] Bessis N Boissier MC Ferrara P Blankenstein T Fradelizi D Fournier C Attenuation of collagen-induced arthritis in mice by treatment with vector cells engineered to secrete interleukin-13 Eur J Immunol 1996 15 2399 2403 10.1002/eji.1830261020 8898952

[B42] Jung S Park YK Lee H Shin JH Lee GR Park SH TGF-beta-treated antigen presenting cells suppress collagen-induced arthritis through the promotion of Th2 responses Exp Mol Med 2010 15 187 194 10.3858/emm.2010.42.3.019 20164680 PMC2845003

[B43] Takayanagi H New immune connections in osteoclast formation Ann N Y Acad Sci 2010 15 117 123 10.1111/j.1749-6632.2009.05303.x 20392226

[B44] Kreymborg K Etzensperger R Dumoutier L Haak S Rebollo A Buch T Heppner FL Renauld JC Becher B IL-22 is expressed by Th17 cells in an IL-23-dependent fashion, but not required for the development of autoimmune encephalomyelitis J Immunol 2007 15 8098 8104 18056351 10.4049/jimmunol.179.12.8098

[B45] Weaver CT Hatton RD Mangan PR Harrington LE IL-17 family cytokines and the expanding diversity of effector T cell lineages Annu Rev Immunol 2007 15 821 852 10.1146/annurev.immunol.25.022106.141557 17201677

[B46] Langrish CL Chen Y Blumenschein WM Mattson J Basham B Sedgwick JD McClanahan T Kastelein RA Cua DJ IL-23 drives a pathogenic T cell population that induces autoimmune inflammation J Exp Med 2005 15 233 240 10.1084/jem.20041257 15657292 PMC2212798

[B47] Trombone AP Claudino M Colavite P de Assis GF Avila-Campos MJ Silva JS Campanelli AP Ibanez OM De Franco M Garlet GP Periodontitis and arthritis interaction in mice involves a shared hyper-inflammatory genotype and functional immunological interferences Genes Immun 2010 15 479 489 10.1038/gene.2010.13 20428191

[B48] Graves DT Fine D Teng YT Van Dyke TE Hajishengallis G The use of rodent models to investigate host-bacteria interactions related to periodontal diseases J Clin Periodontol 2008 15 89 105 10.1111/j.1600-051X.2007.01172.x 18199146 PMC2649707

[B49] Park JC Su C Jung IH Choi SH Cho KS Kim CK Park YB Lee SK Kim CS Mechanism of alveolar bone loss in a collagen-induced arthritis model in mice J Clin Periodontol 2011 15 122 130 10.1111/j.1600-051X.2010.01645.x 21062340

[B50] Ramamurthy NS Greenwald RA Celiker MY Shi EY Experimental arthritis in rats induces biomarkers of periodontitis which are ameliorated by gene therapy with tissue inhibitor of matrix metalloproteinases J Periodontol 2005 15 229 233 10.1902/jop.2005.76.2.229 15974846

[B51] Queiroz-Junior CM Madeira MF Coelho FM Costa VV Bessoni RL Sousa LF Garlet GP Souza Dda G Teixeira MM Silva TA Experimental arthritis triggers periodontal disease in mice: involvement of TNF-alpha and the oral microbiota J Immunol 2011 15 3821 3830 10.4049/jimmunol.1101195 21890656

[B52] Scher JU Ubeda C Equinda M Khanin R Buischi Y Viale A Lipuma L Attur M Pillinger MH Weissmann G Littman DR Pamer EG Bretz WA Abramson SB Periodontal disease and the oral microbiota in new-onset rheumatoid arthritis Arthritis Rheum 2012 15 3083 3094 10.1002/art.34539 22576262 PMC3428472

[B53] Smit MD Westra J Vissink A Doornbos-van der Meer B Brouwer E van Winkelhoff AJ Periodontitis in established rheumatoid arthritis patients: a cross-sectional clinical, microbiological and serological study Arthritis Res Ther 2012 15 R222 10.1186/ar4061 23075462 PMC3580533

